# Lichenoid Drug Eruption Secondary to Adalimumab: A Case Report

**DOI:** 10.7759/cureus.64013

**Published:** 2024-07-07

**Authors:** Asma Alkheraiji, Hend Alotaibi, Husna Irfan Thalib

**Affiliations:** 1 College of Medicine: Dermatology, Majmaah University, Riyadh, SAU; 2 Dermatology, King Saud Medical City, Riyadh, SAU; 3 College of Medicine: General Medicine and Surgery, Batterjee Medical College, Jeddah, SAU

**Keywords:** skin lesions, tnf inhibitors, lichenoid drug eruption, lichen planus, adalimumab

## Abstract

Adalimumab, an anti-tumor necrosis factor-α (TNF-α), is widely prescribed for many autoimmune diseases and chronic inflammatory skin diseases such as hidradenitis suppurative, psoriasis, etc. We report a case of lichenoid drug eruption secondary to adalimumab, a rare side effect, in a 62-year-old female with ulcerative colitis. The skin eruption appeared two weeks after initiating adalimumab. A skin biopsy was taken, and the histopathological findings correlated with a lichenoid drug eruption. Although rare, drug-induced lichen planus has been associated with adalimumab. Early recognition and a high index of suspicion are key in the prompt management of these cases. The discontinuation of adalimumab must be carefully weighed against its therapeutic benefits, as the discontinuation might trigger a severe form of inflammation in the primary autoimmune disease being treated. Extreme caution, early intervention, and a multidisciplinary approach are best for the overall well-being and optimal care of the individual.

## Introduction

Adalimumab is a fully humanized anti-tumor necrosis factor-α (TNF-α) monoclonal IgG1 antibody (mAb), and it is approved by the Food and Drug Administration for the management of many inflammatory autoimmune diseases [1]. Lichenoid drug eruption or drug-induced lichen planus is an uncommon adverse effect of adalimumab, a TNF- α inhibitor [2]. It is clinically identical to idiopathic lichen planus [3]. However, history, clinical examination, and histopathology assist in the diagnosis and differentiation between drug-induced lichen planus and idiopathic lichen planus. We report a case of drug-induced lichen planus secondary to adalimumab.

## Case presentation

A 62-year-old Saudi female presented to the clinic for the evaluation of multiple skin lesions of less than a month's duration. She was a known case of hepatitis B on entecavir treatment for the past two years. She also had ulcerative colitis for 13 years and was taking adalimumab, 40 mg subcutaneous (SC) injection, every two weeks for one month (started with a loading dose of 80 mg SC in the first week followed by 40 mg SC; the week after, she continued with the regular dose). These lesions appeared two weeks after adalimumab was initiated. Few unilateral, itchy, well-defined skin lesions appeared over the right leg and thigh. The smaller lesions resolved with residual pigmentation, however, larger lesions persisted. Initially, she was diagnosed with discoid eczema and, accordingly, she was prescribed betamethasone valerate with mild improvement.

**Figure 1:**
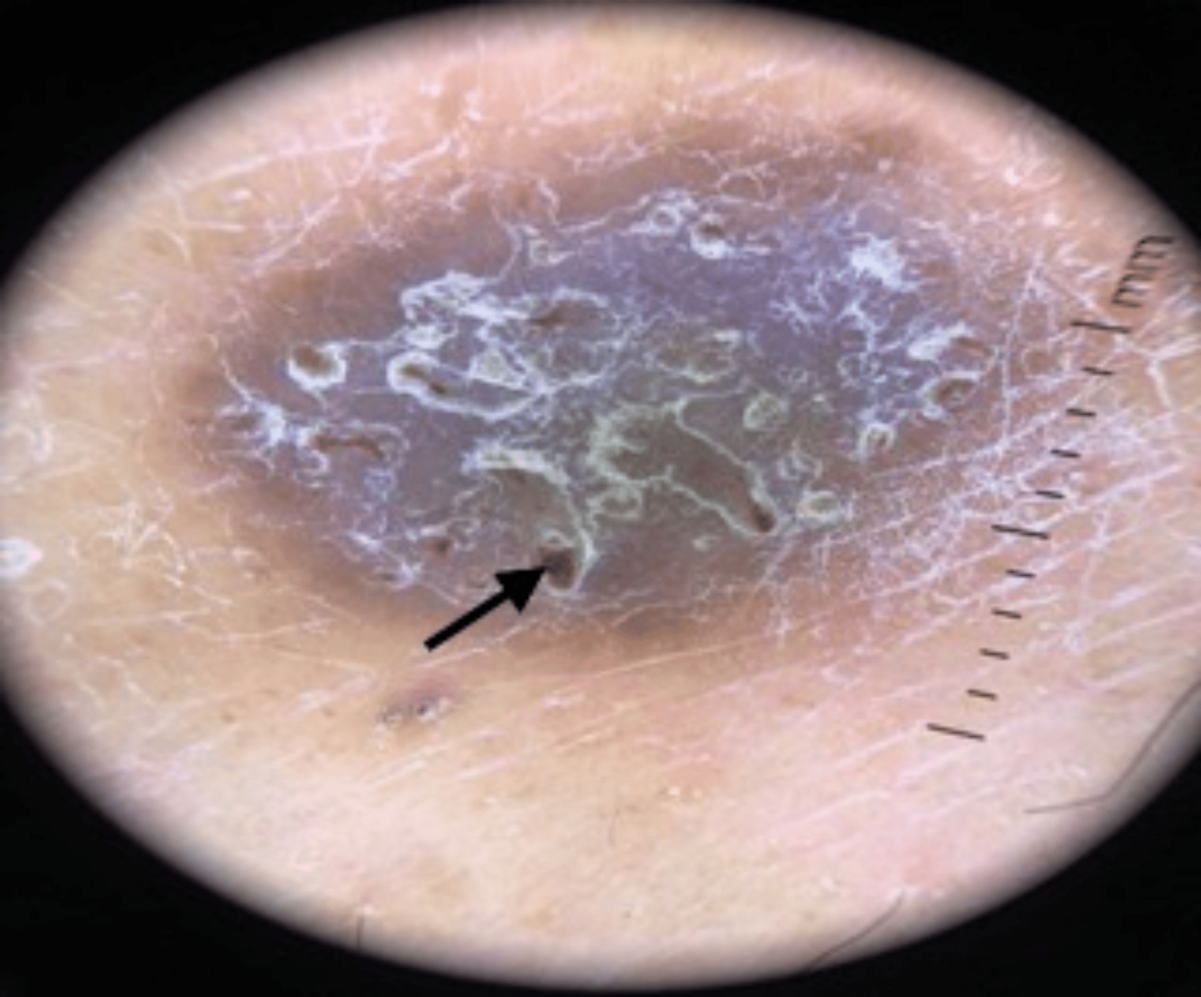
Dermoscopy The dermoscopy image shows a mixture of dark brown, black, and violaceous hues with irregular reticular patterns (indicated by the black arrow), whitish streaks, and a scaly, crusty surface, with indistinct and irregular borders blending into the surrounding skin.

On further examination, a few, small, unilateral, well-defined, hyperkeratotic, scaly plaques that were violaceous in color were visualized over the lateral aspect of the right leg and thigh. Additionally, multiple .5 x 5 cm well-defined rounded brownish hyperpigmented macules were noted over the right forearm. No oral, mucosal, hair, or nail lesions were present. Dermoscopy is shown in Figure 1.

Histological examination

**Figure 2:**
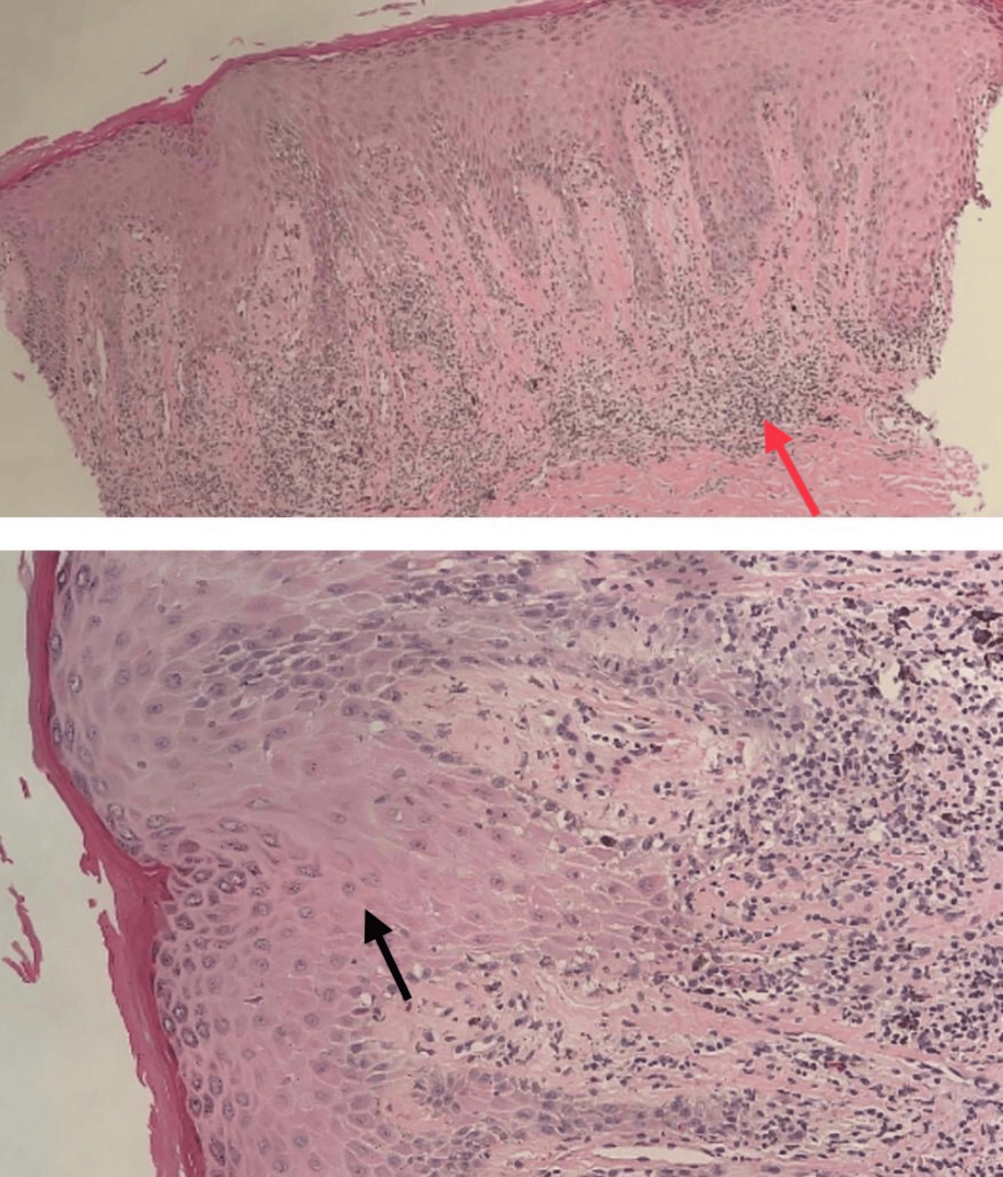
Histopathological examination results Histopathological findings showed orthokeratosis, hypergranulosis (indicated by the black arrow), and acanthosis with an elongation of rete ridges. Lymphocytic infiltrates (indicated by the red arrow) were also noted at the dermo-epidermal junction with necrotic keratinocytes and scattered eosinophils. These findings are suggestive of lichenoid drug eruption (H&E, 100x). Focused view showing lymphocytic infiltrates (H&E, 400x).

Histopathological findings showed orthokeratosis, hypergranulosis, and acanthosis with an elongation of rete ridges. Lymphocytic infiltrates were also noted at the dermo-epidermal junction with necrotic keratinocytes and scattered eosinophils. These findings are suggestive of lichenoid drug eruption. Figure 2 demonstrates the histological examination results.

Serology

During further examination of the patient, serological tests were carried out to rule out other autoimmune diseases and infectious etiologies. Serological investigations, including a complete blood picture, liver function test, and thyroid function test, were within normal limits.

Antinuclear antibody (ANA) 1 was 320. Anti-gliadin immunoglobulin A (IgA) and IgG were negative. Anti-tissue transglutaminase (TTG) IgA and IgG were negative. Hepatitis B antigen and anti-core antibodies were reactive.

Treatment

The above findings were suggestive of lichenoid drug eruption. The eruption of the skin lesions after initiation of adalimumab and histopathological findings suggestive of lichen eruption indicated a causal correlation between the adalimumab and the eruption of the lichenoid lesions. The mild activity of her skin condition was tolerated by the patient, thereby, stopping adalimumab was not recommended, as the therapeutic benefits needed for ulcerative colitis outweighed the need for discontinuation of adalimumab. Topical betamethasone BID for 1 month was prescribed for her skin lesions, and the patient was advised to continue her regular follow-up to assess if there is an increase in the lesion activity.

## Discussion

The cytokine TNF-α is generated by immune cells and mediates various inflammatory and immunoregulatory processes. It contributes to tissue inflammation in immune-mediated inflammatory diseases, including rheumatoid arthritis, Crohn’s disease, and ulcerative colitis [4]. Adalimumab is one of the TNF inhibitors. It suppresses the activity of the inflammatory process and decreases inflammation in the body [1]. It is also a potent and well-tolerated drug; however, it can sometimes produce an uncontrolled release of interferon-α that might induce an autoimmune cutaneous phenomenon [5]. Common cutaneous manifestations caused by adalimumab that were reported in the literature are urticaria, stomatitis, and rashes while more severe side effects include lichenoid eruption, lupus erythematosus, necrotizing vasculitis, and bullous skin lesions [6,7]. Drug-induced lichen planus has been associated with various drugs, and many cases have been reported in the literature. A few cases were also reported where the causative drug was adalimumab. For instance, two cases of mucosal and mucocutaneous lichen planus eruption were reported in patients who were taking adalimumab for psoriasis and one patient with rheumatoid arthritis also developed drug-induced lichen planus three weeks after taking adalimumab [6,8]. In another study, 435 patients who were treated with TNF-α inhibitors for various diseases were included. Out of 435 patients, only 3 developed a lichenoid eruption among which one patient was on adalimumab [9]. Similar cases recently reported have been summarized in Table 1.

**Table 1:** Previous case reports with a similar presentation to our case [10-14]

Study	Patient age/Sex	Drug	Reaction site	Clinical morphology	Time to reaction	Histopathology	Cessation of TNF inhibitor	Outcomes
Markovitz NH. et al. 2022 [10]	53/Female	Casirivimab/ Imdevimab	Right arm, right palm, lower legs	Edematous, well-demarcated, annular, and arcuate erythematous plaques	1 week following treatment, recurred approximately 2 months later	Lichenoid interface dermatitis with occasional eosinophils	Not mentioned	The rash improved with triamcinolone cream and later with clobetasol ointment
Saad et al. 2021 [11]	68/Female	Adalimumab	Forearms	Hypertrophic LP	Several months later	Acanthotic epidermis with orthohyperkeratosis, wedge-shaped hypergranulosis, dermal inflammatory lichenoid infiltrate, and lymphocyte exocytosis in basal layers (H&E, 40×)	No	Slight improvement with topical corticosteroid treatment
Ghiam N et al. 2020 [12]	63/Female	Ixekizumab	Back, buttocks, anterior upper thigh, posterior thighs, posterior legs, and lower abdomen. Linear erosions were noted on the back and the upper extremities	Multiple, generalized, purple, polygonal papules and plaques on the patient's back. The lesions were eczematous and not scaly, consistent with lichen planus.	1 week	Punch biopsy showing hyperkeratosis, focal wedge-shaped hypergranulosis, vacuolar alteration of the basal layer, and band-like superficial inflammatory lymphocytic infiltration, consistent with lichen planus (H&E, 10x)	Yes	Ixekizumab was stopped and triamcinolone acetonide 0.1% topical cream for the body and clobetasol 0.05 % solution for the scalp were prescribed. At the two-week follow-up visit, she had partial resolution of the pruritus and skin lesions. At the six-week follow-up visit, her skin had almost cleared and the pruritus had substantially diminished
Wendling et al. 2013 [13]	47/Male	Adalimumab	Legs, forearms, and the perineal region + oral involvement	Pruriginous shiny, firm, and reddish purple skin lesions	52 months	Histopathological findings consistent with the diagnosis of lichen planus	Yes, change to infliximab	Skin evolution was favorable under topical and oral steroids Improvement
Vergara et al. 2002 [14]	60/Male	Infliximab	Cutaneous arm flexor	Erythematous papules, some polygonal in shape	3 weeks	Necrotic keratinocytes, focal detachment of the dermo-epidermal junction, intense bandlike inflammatory infiltrates of lymphocytes, histiocytic in the superficial dermis	Yes	Complete improvement and recovery

Drug-induced lichen planus and idiopathic lichen planus both exhibit many similar features on clinical examination. Although the drug history of the patient can differentiate between idiopathic lichen planus and lichenoid drug eruption, few findings are specific and evident on histopathological examination. Lichenoid drug eruption is larger, generalized, and photo-distributed; it also spares the classical sites (mainly mucous membranes) of idiopathic lichen planus [3]. Lesions of idiopathic lichen planus are shiny, flat-topped, polygonal, and violaceous, and Wickham’s striae are prominently present [3]. Drug-induced lichen planus shows more distinct desquamation with psoriasis-like or eczema-like morphology [8]. Histopathologically, parakeratosis, eosinophils, plasma cells, and some deep perivascular and peri-adnexal infiltrates are observed, which are not present in cases of idiopathic lichen planus [7].

Patients mostly recall the time interval between the skin lesion eruption and the start of the offending drug, which varies from a few weeks to months to a year or more depending upon the drug class, dose, concurrent medications, and host reaction [15]. Our patient developed skin lesions after two weeks of the initiation of adalimumab.

Another diagnostic criterion is the disappearance of skin eruption after the cessation of adalimumab [2]. Patients start improving after a few months of cessation of adalimumab. Asarch et al. reported a complete recovery of seven patients out of nine while the remaining two patients showed mild to moderate improvement when they stopped taking TNF-α blockers [8]. However, in another report, a patient did not improve after the discontinuation of the drug [16]. Treatment of the lichenoid drug eruption includes discontinuation of the offending drug and the use of glucocorticoids [17]. Skin lesion resolution after the removal of the offending agent also differs from patient to patient and is reported to range from a few weeks to a year [18]. In some cases, the reaction is so severe that the discontinuation of adalimumab is the only treatment, but in other cases, the reaction is milder and there is no need for the withdrawal of the drug.

## Conclusions

In conclusion, TNF-α blockers are approved to treat a variety of diseases like rheumatoid arthritis, psoriasis, juvenile idiopathic arthritis, and inflammatory bowel disease (Crohn’s disease and ulcerative colitis). Adalimumab-induced lichen planus is rare, and the incidence is uncommon. Differentiation between lichenoid drug eruption and idiopathic lichen planus is based on clinical and histopathological findings. It is important when using adalimumab to ensure prompt diagnosis of any side effects and the initiation of the appropriate treatment on a case-to-case basis. In addition, before the discontinuation of adalimumab, it is necessary to weigh the benefits of the drug on the primary disease. As in our case, the patient with ulcerative colitis responded well with adalimumab where other treatments failed and the skin eruptions were mild and tolerable.
